# Supplementary material for: Plasticity of Expression of Stem Cell and EMT Markers in Breast Cancer Cells in 2D and 3D Culture Depend on the Spatial Parameters of Cell Growth; Mathematical Modeling of Mechanical Stress in Cell Culture in Relation to ECM Stiffness
Source: Bioengineering (Basel). 2025 Feb 4;12(2):147. doi: 10.3390/bioengineering12020147 (PMC11852359; doi:10.3390/bioengineering12020147)
Supplement: Supplementary file 1 [file bioengineering-12-00147-s001.zip › bioengineering-3403305-supplementary.pdf]

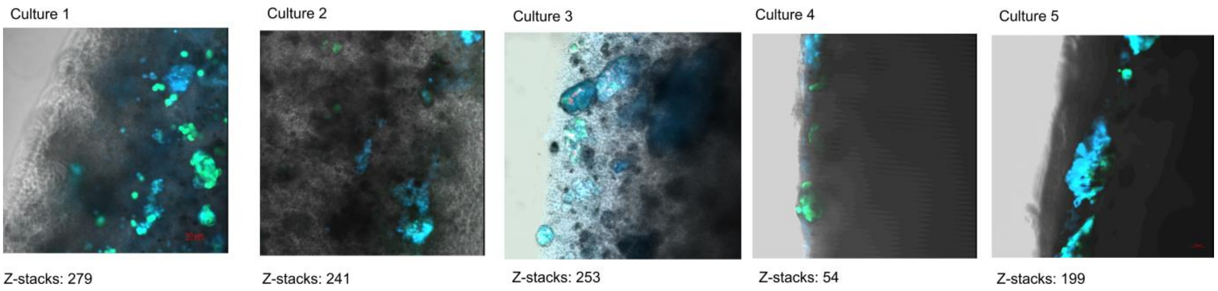

Figure S1. Images of selected 3D bioprinted cultures (single confocal plane, number of Z-stacks under each image). Staining: calcein, Hoechst 33342. Culture 1 was used for modeling.
